# Novel Molecular Hallmarks of Group 3 Medulloblastoma by Single-Cell Transcriptomics

**DOI:** 10.3389/fonc.2021.622430

**Published:** 2021-03-18

**Authors:** Chaoying Qin, Yimin Pan, Yuzhe Li, Yue Li, Wenyong Long, Qing Liu

**Affiliations:** Department of Neurosurgery in Xiangya Hospital, Central South University, Changsha, China

**Keywords:** group 3 medulloblastoma, single-cell sequencing, hallmark, prognosis, molecular cascades

## Abstract

Medulloblastoma (MB) is a highly heterogeneous and one of the most malignant pediatric brain tumors, comprising four subgroups: Sonic Hedgehog, Wingless, Group 3, and Group 4. Group 3 MB has the worst prognosis of all MBs. However, the molecular and cellular mechanisms driving the maintenance of malignancy are poorly understood. Here, we employed high-throughput single-cell and bulk RNA sequencing to identify novel molecular features of Group 3 MB, and found that a specific cell cluster displayed a highly malignant phenotype. Then, we identified the glutamate receptor metabotropic 8 (GRM8), and AP-1 complex subunit sigma-2 (AP1S2) genes as two critical markers of Group 3 MB, corresponding to its poor prognosis. Information on 33 clinical cases was further utilized for validation. Meanwhile, a global map of the molecular cascade downstream of the MYC oncogene in Group 3 MB was also delineated using single-cell RNA sequencing. Our data yields new insights into Group 3 MB molecular characteristics and provides novel therapeutic targets for this relentless disease.

## Introduction

Medulloblastoma (MB) is one of the most prevalent malignant (WHO IV) brain tumors in children, accounting for 15–20% of pediatric central nervous system tumors (1). Unfortunately, over 40% of patients with MB are diagnosed with metastases, with a grim median survival (2–4). Multimodal therapy, including combination of surgical resection, radiation, and adjuvant chemotherapy, has become a standard for MB, even though approximately one-third of patients with MB die from the disease (5). Thus, the identification of critical regulators that control MB malignance could facilitate the development of more effective therapeutics.

Current consensus identifies the existence of four major MB subgroups (Sonic Hedgehog [SHH], Wingless [WNT], Group 3, and Group 4) with different molecular characteristics. Group 3 MB is refractory to intensive multimodal therapy and displays the worst prognosis. However, the molecular characterization of Group 3 MB remains largely unknown, even less than the cells of origin. In contrast to WNT and SHH MBs, Group 3 tumors contain fewer nucleotide variants and germline mutations (6–9). Previous studies demonstrated that a subset of Group 3 tumors exhibits overexpression of transcription factors of the growth factor independent 1 family as a result of DNA structural changes that transform the genes encoding these factors almost into super enhancers (10). Pathway analysis indicated that transforming growth factor beta signaling pathways are also activated in Group 3 MB. However, the significance of MB tumorigenesis remains to be determined (7, 10, 11). Currently, the most validated prognostic marker is *MYC* oncogene amplification, found in approximately 20% of patients with Group 3 MB. However, how *MYC* drives tumorigenesis in a subset of Group 3 tumor cells remains to be defined.

Systematic search and analysis using multi-omics sequencing showed that MB is highly heterogeneous with intratumoral and intertumoral heterogeneity. Recently, single-cell transcriptomic methods have been utilized (12–16) to resolve tumor heterogeneity (17, 18), reconstruct tumor lineages (19–21), explore rare subpopulations (22, 23), and provide insights into the phenotypes of stromal and tumor cells in different cancers (24–26). It has been documented that single-cell sequencing could remove the barriers that previously challenged bulk genomic studies of patients with Group 3 MB, and hopefully, unearth Group 3 MB applicable molecular hallmarks and pathways for clinical utilization, similarly to the isocitrate dehydrogenase (IDH) and BRAF V600E mutations for glioblastoma.

In this study, we took advantage of one cohort (GSE119926) of scRNA-seq data composed of 25 tumor samples and 11 patient-derived xenograft (PDX) models (27). A total of 2762 cells were gathered from surgically removed Group 3 MB scRNA-seq data of eight patients according to their matching subtype information. Through detailed analysis of cellular heterogeneity, we identified a specific cell cluster with the tumorigenesis signature of Group 3 MB and interrelated molecular cascades initiated by MYC. The marker genes of this specific cluster were selected, followed by validation and selection, performed with tumor samples from 33 patients with Group 3 MB. Consequently, we unearthed the GRM8 gene, encoding metabotropic glutamate receptor 8 (MGLUR8), a G-protein coupled glutamate receptor reported to significantly influence the risk of central nervous system (CNS) disease (28–30), and the AP1S2 gene, encoding AP-1 complex subunit sigma-2, a component of adaptor protein complex 1 and correlating with CNS disorder (31), as novel hallmarks linked to poor prognosis of Group 3 MB. Thus, our findings identified GRM8 and AP1S2 as potential targets for treatment of patients with MYC+ Group 3 MB in the future.

## Materials and Methods

### Patient Selection and Data Preprocessing

For MB single-cell transcriptome expression data, we searched in the Gene Expression Omnibus (GEO) and finally included one cohort (GSE119926) with scRNA-seq data of 25 tumor samples and 11 patient-derived xenograft (PDX) models for downstream analysis (27). According to its matching subtype information, normalized scRNA-seq data of eight patients with Group 3 MB (MUV11, SJ17, SJ917, SJ617, MUV29, BCH1205, MUV34, and BCH825) were extracted from the original downloaded scRNA-seq expression matrix. In total, after removing data with <2500 gene expression, we obtained an scRNA-seq expression matrix of 2762 cells from the surgically removed Group 3 MB sample in our study. For bulk transcriptome expression data, we applied Gliovis (gliovis.bioinfo.cnio.es), a web tool collecting brain tumor sequencing data from GEO, and the Cancer Genome Atlas database (32) to search for bulk data with MB clinical information and follow-up data. We included two MB cohorts (Cavalli et al., n = 763 and Griesinger et al., n = 130) in our study (33, 34). These two MB cohorts were sequenced by Affymetrix arrays (HG-U133_Plus_2, HG-U133A, HG_U95Av2, and HuGene-1_0-st), normalized by a multi-array average method using R package “affy.” For genes with several probe sets, we chose the median value as the ultimate expression level. Using function “Combat” in R package sva, the batch effect produced by technical biases during the sequencing process was removed to reduce its side effects on downstream analysis.

### Single-Cell Sequencing Data Analysis

For the obtained scRNA-seq data of Group 3 MB, R package “seurat” was applied for initial normalization and an unsupervised clustering process was subsequently performed (35). Then, the function “Find Variable Features” in Seurat was performed to find genes with high variability for downstream analysis, choosing the top 2000 genes with high standardized variance. Through integration of principle components analysis and t-distributed stochastic neighbor embedding (t-SNE), we reduced the dimension of expression data and divided 2762 cells of Group 3 MB into clusters with distinct expression patterns. Simultaneously, marker genes for each cluster were found according to its adjusted p-value and average log-transformed fold change value.

### Microarray Data Analysis

Based on R package “limma,” a differentially expression analysis was performed on the normalized microarray data by applying a Bayesian algorithm to find differentially expressed genes between tumor and normal sample, only data with adjusted *p*-value < 0.05 were included in our study for further analysis. According to the corresponding follow-up information, we conducted a Kaplan-Meier survival analysis on the genes of interest.

### Cell Trajectory Analysis

In addition, the R package Monocle was adopted to conduct a time-series analysis of single-cell expression data, which orders every cell in pseudo-time and arranges them along a trajectory corresponding to a biological process such as cell differentiation, without knowing in advance which gene determines that progress (36). The Monocle reduces marker gene expression data of individual clusters from a high dimension form into a low-dimension form through a machine learning algorithm called reversed graph embedding. In that low-dimensional form, each cell is arranged into a branching line according to the sequence of the biological process.

### Gene Set Variation Analysis (GSVA) and GO/KEGG Enrichment Analysis

To compare the enrichment degree of pathways and functions between each cluster, we used a GSVA algorithm, a gene set enrichment (GSE) method that estimates the variation of pathway activity for microarray and transcriptome data, to calculate the GSVA score of each cluster on different gene sets (37). For the calculation of tumor characteristics and pathway enrichment degree, input gene sets were obtained from the molecular signature database (MsigDB) (https://www.gsea-msigdb.org/gsea/msigdb). The gene sets of the KEGG pathway come from the C2 collection (curated gene sets) in MsigDB, and the tumor characteristic gene set comes from the H collection (hallmark gene sets) in MsigDB. By comparing the GSVA scores of each cluster, we could compare the relative enrichment levels of tumor-related pathways or features. In addition, the R package “ClusterProfiler” was used to conduct a hypergeometric distribution test on each cluster’s marker genes to perform GO and KEGG annotation (38).

### Protein-Protein Interaction (PPI) Network Development

Using the STRING database, a PPI network was developed according to the marker genes of each cell cluster. We then utilized Cytoscape to rearrange the PPI network downloaded for the STRING database according to its interaction characteristic (39). In Cytoscape, the CytoHubba plug-in was used to calculate and rank the interaction degree between downstream proteins of marker genes. Additionally, according to the interaction degree, we adjusted the color and position of the protein node, turning the highest degree node darker and placing it in the center.

### Immunohistochemistry

Tumor tissues from 33 patients with Group 3 MB were perfused with 4% paraformaldehyde and fixed in 10% neutral buffered formalin mixed with 70% ethanol. Immunohistochemistry staining was performed according to protocols from Cell Signaling Technology. The antibodies used for immunostaining were Anti-MAGP1 (encoded by MFAP2, ab231344, Abcam) and Anti-MGLUR8 (encoded by GRM8, ab176301, Abcam). Quantification of mean fluorescence intensity was achieved using Image-Pro-Plus software.

### Real-Time Quantitative PCR

RT-qPCR was performed using total RNA from the central tissues of eight (Numbered 1–8) patients with normal brain tissues (1–2), WNT/SHH (3–5), and Group 3 (6–8) MB. Total RNA was extracted using TRI Reagent (Molecular Research Center, Inc.) according to the manufacturer’s protocol, and cDNA was synthesized using random hexamer and oligo (dT) primers using Thermo ScriptTM RT-PCR (Invitrogen). The gene-specific primers employed were purchased from the NDT Corporation. PCR was performed for 40 cycles of 95 °C for 15 s and 60 °C for 30 s. H-actin was amplified as a control (Forward: ACCCTGAAGTACCCCATCGAG; reverse: AGCACAGCCTGGATAGCAAC). Specific expression of MFAP2 and GRM8 in cell lines was established using total RNA obtained from tumor tissues and amplified with primers for each one (MAGP: Forward, CAGTCCCAGCAGCAAGTCCA and Reverse, AAGCAGACCTCGTTGAGACAC; GRM8: Forward, ACCTGCATCATTTGGTTAGCTT, and Reverse, AAACCTTGGGCATATAGAGCA) using SYBR Green PCR Master Mix (ThermoFisher Scientific).

### Statistical Analysis

The correlation coefficient between the IOD area and numerical variables including age (year), tumor size (mm^3^), and Ki67 (%) was separately calculated using the Spearman and Pearson correlation analysis. Random Grouping t-test was used to assess the relevance between the IOD area and binary clinical information including sex, cystic change, hydrocephalus, while one-way ANOVA was used to compare the differences among multi-grouped variables including tumor location. In addition, we applied Kaplan-Meier survival analysis and log-ranked test to conduct a survival comparison, where the median value was implemented to cut the relative genes expression level into high and low Groups. All the above-mentioned statistical analyses were conducted using R software (version 3.6.0). All *p*-values < 0.05 were considered statistically significant.

## Results

### Landscape of Cellular Heterogeneity at Single-Cell Level Within Group 3 MB

**Figure 1:**
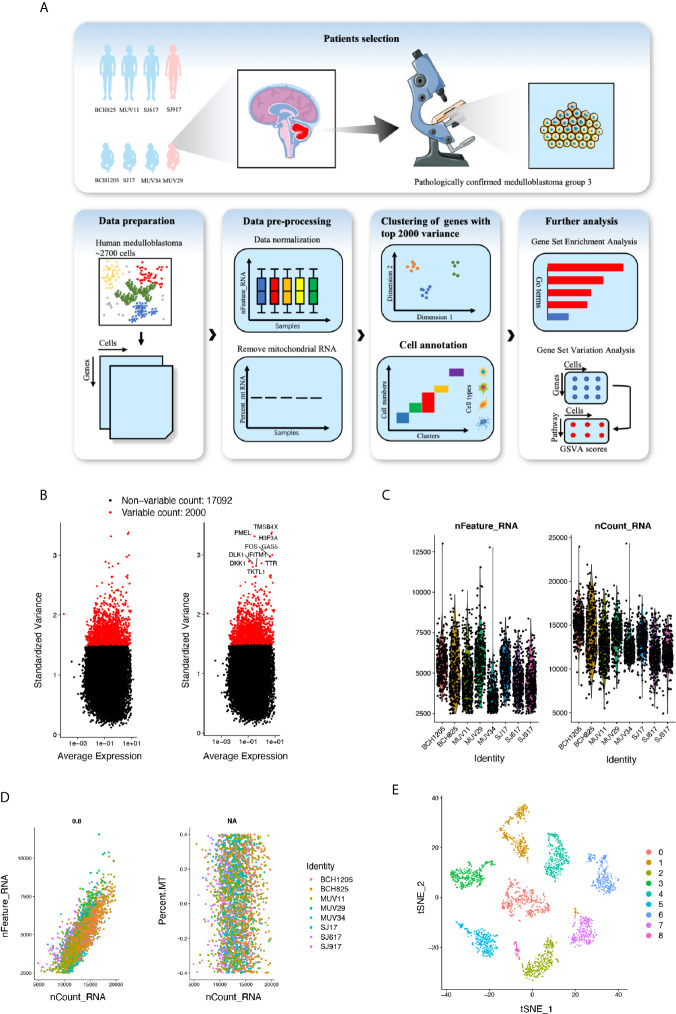
**(A)** Overall data analysis process of single-cell transcriptome expression landscape of Group 3 medulloblastoma (MB) (n=8). **(B)** Genes with top 2000 standard variance selected for subsequent analysis. **(C)** Violin plot of RNA features, and RNA counts in patients with MB. **(D)** Scatter plot of expression level of mitochondrial RNA, RNA features and RNA counts. **(E)** T-distributed stochastic neighbor embedding (t-SNE) plot of Group 3 MB cells revealing 0–8 cell clusters.

Identification of critical cell clusters regulating cancer initiation and progression may help develop novel and effective strategies to overcome the treatment resistance associated with Group 3 MB. Thus, we initially selected the MB single-cell RNA sequencing (seq) datasets published in the GEO and finally included one cohort (GSE119926) of 25 tumor samples and 11 patient-derived xenograft (PDF) models for downstream analysis. According to its matching subtype information, normalized scRNA-seq data of eight patients with Group 3 MB (MUV11, SJ17, SJ917, SJ617, MUV29, BCH1205, MUV34, and BCH825) were extracted from the original scRNA-seq expression matrix, including a total of six male patients (three adults and three children) and two female patients (one adult and one child) (**Figure 1A**). A total of 2762 single-cell datasets of the above eight patients were organized into an expression matrix. Data preprocessing was initiated by normalizing the RNA expression of each cell and then removing mitochondrial RNAs. All single cells were divided into individual clusters according to their distance distribution after dimensionality reduction through t-SNE, gene set enrichment analysis, and functional annotations such as GSEA and GSVA scores on specific cell clusters was performed (**Figure 1A**). The normalization for the expression matrix was initiated by calculating the standard deviation of the gene expression in 2762 cells. We selected 2000 sufficient genes with a high standard deviation (**Figure 1B**). The normalized gene type (n-Feature-RNA), gene counts (n-Count-RNA), and mitochondrial gene numbers (MT-RNA) of each patient’s tumor cells (mitochondrial genes were previously removed) were plotted, revealing that RNA levels in each of the patient’s tumor cells were expressed in a similar scope without apparent dispersion (**Figure 1C**). Notably, the gene sequencing depth was found to be consistent with the scatter diagram since the relationship between the number of gene types and the number of counts was positively correlated (**Figure 1D**). To further explore the expression feature of the scRNA data, we initially normalized the gene expression matrix and selected characteristic genes with a high standardized deviation for downstream analysis. After principle components analysis (PCA) and identification of distinct principle components, the representative principle components were chosen subsequently for unsupervised clustering process. Finally, we conducted a clustering using t-distributed stochastic neighbor embedding (t-SNE) and divided the cells into nine individual cell clusters (Numbered 0–8) with various differentiation features. Finally, we divided the cells into nine individual cell clusters (Numbered 0–8) with various differentiation features (**Figure 1E**). These data suggest that Group 3 MB is highly heterogeneous with different cell subgroups and genetic characteristics.

### Cell Clusters Reflect Transcriptional Heterogeneity and Trajectory

**Figure 2:**
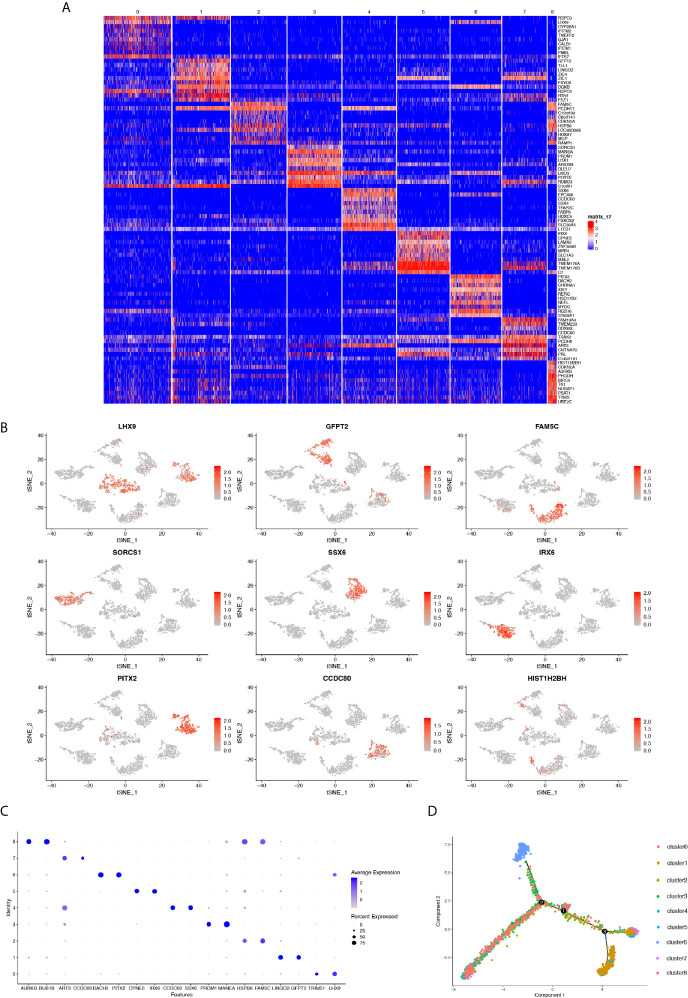
**(A)** Heatmap showing the genes differentially expressed in each cluster. **(B)** t-SNE plots of marker gene expression levels in each cluster. **(C)** Bubble plot depicting the expression level of selected genes in each cluster. Color depth represents average expression level and bubble size the percentage of expression. **(D)** Cell trajectory analysis showing five main cell branches.

We next sought to investigate the transcriptional heterogeneity of cells in each cluster by depicting the gene expression profile using a heatmap that showed distinguishable differences in gene expression preferences in each cluster. Except for cluster 0, we identified significantly upregulated genes in clusters different from each other, indicating the transcriptional heterogeneity of the nine clusters (**Figure 2A**). Then, we performed scatter plots by t-SNE colored by the expression of a single gene in all nine cell clusters. The genes showing the highest expression level in each individual cluster were selected for mapping. Consistently, these genes were only specifically expressed in their own cluster (**Figure 2B**). Moreover, the bubble graph was also plotted to further identify the genes that were most upregulated. Notably, the bubble color refers to the average gene expression in all cells of each cluster, whereas bubble size represented the gene expression percentage in the cluster. The top genes containing the highest level of the above two criteria in each cluster were emphasized with a large size and darker color (**Figure 2C**). Due to transcriptional heterogeneity among the nine cell clusters, we speculated that single-cell RNA-seq may uncover the heterogeneity of biological features correlated with MB formation. To test our hypothesis, the R package Monocle was adopted to conduct a time-series analysis of the single-cell expression data to address this point. We ordered every cell in pseudo-time and arranged them along a trajectory corresponding to a biological process such as cell differentiation, without knowing in advance which gene determines that progress (36). Each cell of the nine clusters was arranged into a branching line according to the sequence of the biological process (**Figure 2D**). We observed that cluster 6 (colored blue) was relatively isolated at the beginning of a differentiation tree at point “A,” indicating that cell cluster 6 may play a key role in cancer initiation and progression.

### Annotation of the Tumorigenesis Featured Cluster and Its Downstream Cascades

**Figure 3:**
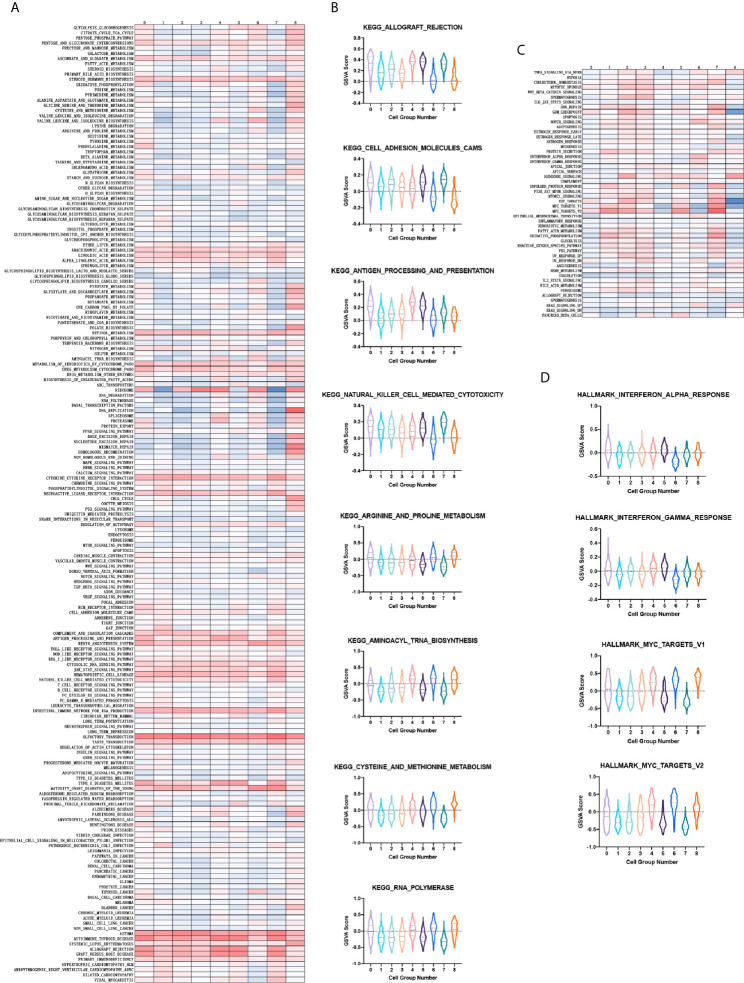
**(A)** Heatmap showing the gene set variation analysis (GSVA) scores of each cell cluster in the KEGG pathway. **(B)** Violin plot depicting the GSVA score of specific KEGG pathways in each cell cluster. **(C)** Same as A in H collection in molecular signature database (MsigDB). **(D)** Same as B in H collection in MsigDB.

With regards to the aforementioned feature of cluster 6, we next sought to verify its exclusive tumorigenesis feature among the nine cell clusters. To compare the enrichment degree of pathways and functions among each cluster, we used the GSVA and GSE methods to estimate the variation in pathway activity from microarray and transcriptome data, to calculate the GSVA score of each cluster on different gene sets (37). For the calculation of tumor characteristics and pathway enrichment degree, the input gene sets were obtained from the molecular signature database (MsigDB). Among them, the KEGG pathway gene sets derived from the C2 collection (curated gene sets) (**Figure 3A**). Further, we performed a violin plot by selecting the pathways demonstrating specific up- or down-regulation in cluster 6 compared to the others, quantified by the GSVA score. The degree of enrichment of cluster 6 in some tumor-related pathways was significantly higher than that of the other clusters, such as cell adhesion molecules cams, and arginine and proline metabolism for metastasis of tumors (40). Nevertheless, in immune-related pathways such as antigen procession and presentation, and natural killer cell mediated cytotoxicity which have been correlated to antitumor cytotoxicity, the enrichment was significantly lower (**Figure 3B**). Consistently, tumor characteristic genes set from H collection (hallmark gene sets) in MsigDB (**Figure 3C**) revealed that the degree of enrichment of cluster 6 in MYC targets V1/V2 was remarkably higher than that of other clusters identified as critical for Group 3 MB. The enrichment of antitumor cytotoxicity in cluster 6, such as interferon alpha response and interferon gamma response, were much lower than those of other clusters (**Figure 3D**). Collectively, these results demonstrated the pivotal role of cluster 6 in the malignant behavior of Group 3 MB and were marked for further validation.

**Figure 4:**
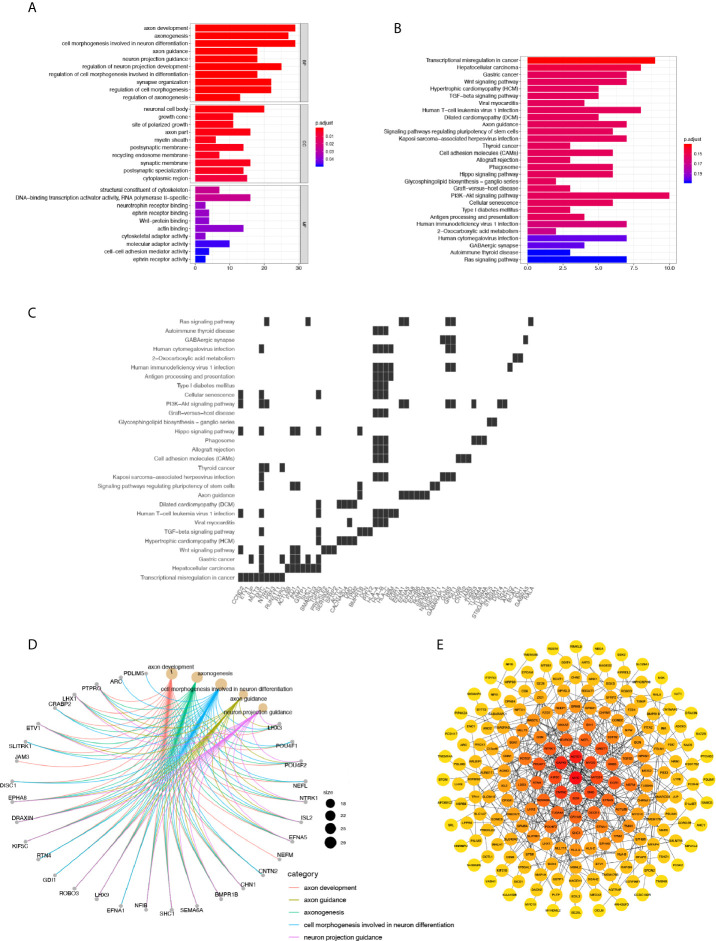
**(A)** GO annotation of cluster 6. **(B)** Gene net plot showing the common genes in multiple GO entries. **(C)** Heatmap showing the genes involved in each pathway. 6. **(D)** KEGG annotation of cluster 6. **(E)** Protein-Protein Interaction network of marker genes in cluster 6, where the node with the highest degree of interaction is darker and located in the center.

To depict the detailed annotation of the signaling pathways involved in cluster 6, we then performed GO enrichment analysis for the marker genes of the six clusters, and suggested that the most active pathways were neuron- and axon-related, such as axon development, axonogenesis, cell morphogenesis involved in neuron differentiation, axon guidance, neuron projection guidance, and regulation of neuron projection development. Meanwhile, genes associated directly or indirectly to these signaling pathways were shown (**Figures 4A, B**). The main pathways for KEGG enrichment in cluster 6 were also analyzed. Interestingly, the top pathways obtaining the highest scores were tumor- or cancer-related, corresponding to previous observations. For example, transcriptional misregulation in cancer, hepatocellular carcinoma, gastric cancer, and the WNT signaling pathway. We also plotted a heatmap of the genes related to these pathways (**Figures 4C, D**). These findings supported our hypothesis that cluster 6 cells were responsible for the tumorigenic character of Group 3 MB. To gain insight into downstream protein interaction and cascades, we constructed a PPI network according to cluster 6 marker genes by using the STRING database. We then utilized Cytoscape to rearrange the PPI network downloaded for the STRING database according to its interaction characteristic (39). In addition, according to the interaction degree, we adjusted the color and position of the protein by turning the highest degree node darker and placing it in the center of the map. Importantly, our data showed that MYC had the highest degree of interaction among all marker genes in cluster 6, consistent with the previous findings that amplification of the *MYC* oncogene is the most common genetic alteration in patients with Group 3 MB (**Figure 4E**). Together, our findings indicated that cluster 6 drives malignancy in Group 3 MB with MYC positivity.

### Identification of Novel Hallmarks in Group 3 MB

**Figure 5:**
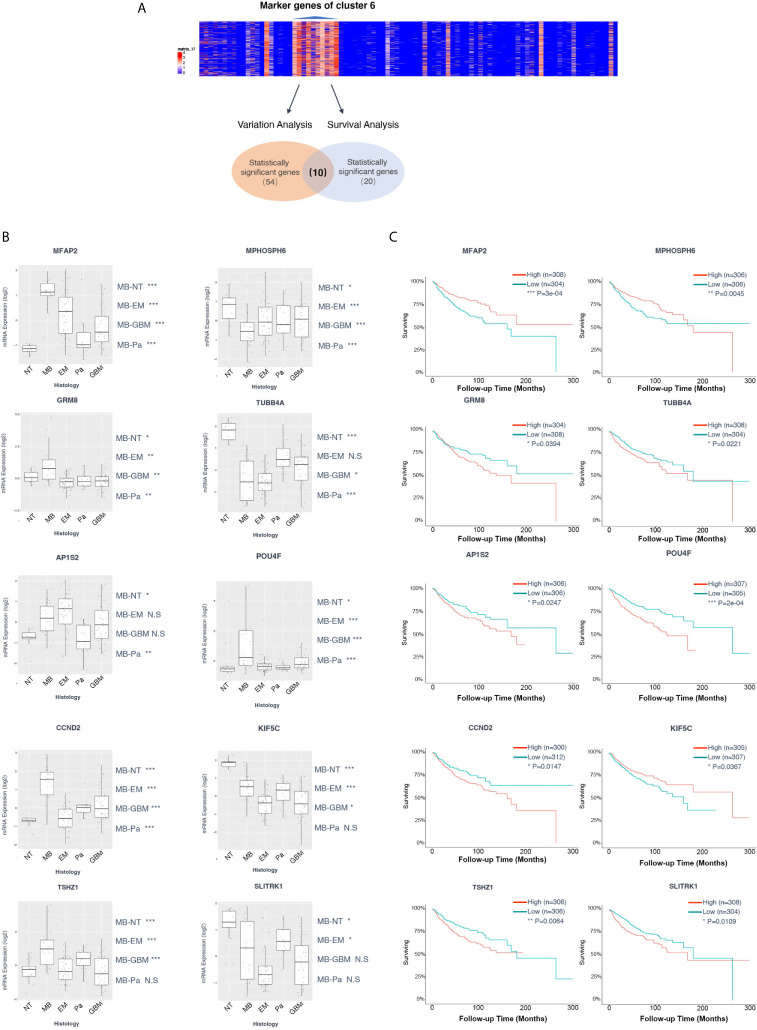
**(A)** Diagram of the screen process of Kaplan-Meier survival and variation analyses for the marker genes of cluster 6 with two cohorts of microarray data. Fifty-four genes showed specific upregulation in one tumor type or normal brain. Twenty genes exhibited correlation with the survival of patients with medulloblastoma (MB). Ten genes contained significant differences of either expression or survival analysis, including TUBB4A, TSHZ1, SLITRK1, POU4F1, MPHOSPH6, MFAP2, KIF5C, GRM8, CCND2, and AP1S2. **(B)** Box plot of variant analysis of microarray data of the MB patient cohort grouped showing expression levels of TUBB4A, TSHZ1, SLITRK1, POU4F1, MPHOSPH6, MFAP2, KIF5C, GRM8, CCND2, and AP1S2 in various brain tumors and normal brain. NT, normal tissue; MB, medulloblastoma; EM, ependymoma; Pa, pilocytic astrocytoma; GBM, glioblastoma. **(C)** Kaplan-Meier survival curve of microarray data of the MB patient cohort grouped by TUBB4A, TSHZ1, SLITRK1, POU4F1, MPHOSPH6, MFAP2, KIF5C, GRM8, CCND2, and AP1S2 expression level. Data are mean ± s.e.m. *P ≤ 0.05; **P ≤ 0.01; ***P ≤ 0.001; ****P < 0.0001; NS, not significant (P > 0.05).

Given the stepwise dissection of cluster 6 in Group 3 MB, the malignance signature of this cluster became relatively clear. We selected a set of microarray data from a cohort consisting of common pediatric malignant brain tumors containing 15 pilocytic astrocytomas, 46 ependymomas, 20 glioblastomas, 22 MBs, and 13 non-tumor brain control samples obtained from epilepsy surgery to generate the bulk transcriptional dataset for differential expression analysis (34) (**Figure 5A**). We performed a variation analysis with normalized microarray data of this mixed pediatric tumor cohort to compare the expression level of cluster 6 marker genes in variant tumor types and normal brain, and found that 54 genes in total showed specific upregulation either in one tumor type or in the normal brain (**Figure 5A**, **Supplementary Materials**). Notably, some genes were specifically upregulated in MB, consistent with their high expression in Group 3 MB in our previous analysis. Meanwhile, another cohort of 763 patients with MB was included to conduct a Kaplan-Meier survival analysis on the marker genes of cluster 6. In total, 20 genes correlated with the survival of patients with MB. By combining these results, 10 genes showed significant differences of either expression levels or survival analysis, including TUBB4A, TSHZ1, SLITRK1, POU4F1, MPHOSPH6, MFAP2, KIF5C, GRM8, CCND2, and AP1S2 (**Figure 5A**). Among them, TSHZ1, MFAP2, GRM8, CCND2, and AP1S2 showed dramatically higher expression levels in MB than other tumors and the normal brain (**Figure 5B**). Of note, the prognosis of the patients highly expressing TSHZ1, GRM8, CCND2, AP1S2 was poorer than low expressing patients, while only MFAP2 showed an adverse correlation between its gene expression and prognosis (**Figure 5C**). Taken together, these data indicate that some marker genes in cluster 6 were truly upregulated in Group 3 MB and affected its prognosis; hence, these five genes were initially identified as potential hallmarks of Group 3 MB.

### GRM8 and AP1S2 Are Hallmarks Indicating Poor Prognosis of Patients With Group 3 MB

**Figure 6:**
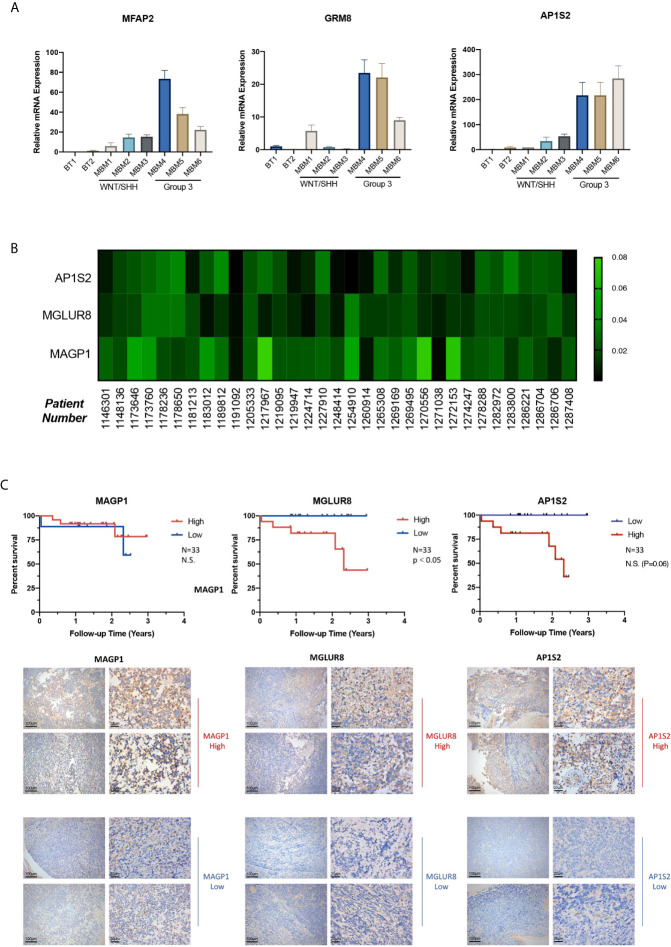
**(A)** GRM8, AP1S2, and MFAP2 mRNA levels of two normal brain tissue obtained from epilepsy surgery (Numbered 1 and 2), three Wingless/Sonic Hedgehog medulloblastoma (MB) (Numbered 3–5), and three Group 3 MB (Numbered 6–8). H-actin was used as the endogenous reference gene. The expression level is presented with graphs. **(B)** Heatmap of Immunohistochemistry staining with (GRM8 encoding), (AP1S2 encoding), and (MFAP2 encoding) antibodies for the 33 Group 3 MB. Protein IOD/Are is represented by the indicated color. **(C)** Images of immunohistochemistry staining showing high and low expression of MGLUR8 (GRM8 encoding), AP1S2 (AP1S2 encoding), and MAGP1 (MFAP2 encoding) in the series of Group 3 MB patients (10X and 40X magnification). Kaplan-Meier survival curve of the 33 patients with Group 3 MB grouped by expression level of GRM8, AP1S2, and MFAP2 encoding. Data are mean ± s.e.m. ****P < 0.0001; NS, not significant (P > 0.05).

Based upon the stepwise bioinformatic analysis with single-cell and bulk datasets, we narrowed down the pool of Group 3 MB’s potential hallmarks. However, for further validation a biochemical verification was needed; therefore, we performed quantitative PCR with samples from two normal brain tissues obtained from epilepsy surgery (Numbered 1 and 2), three WNT/SHH MBv (Numbered 3–5), and three Group 3 MB (Numbered 6–8) to test the gene expression levels of TSHZ1, MFAP2, GRM8, CCND2, and AP1S2. Among them, MFAP2, GRM8, and AP1S2 were highly expressed in patients 6, 7, and 8, diagnosed with Group 3 MB through pathological tests (**Figure 6A**), while TSHZ1 and CCND2 did not exhibit the same expression trends (**Supplementary Materials**). This result supported the provisional exclusion of these two genes and to focus on MFAP2, GRM8, and AP1S2. Then, with samples of 33 patients pathologically diagnosed with Group 3 MB, we performed immunohistochemistry (IHC) staining to test the expression level of MAGP1 encoded by MFAP2, MGLUR8 encoded by GRM8, and AP1S2 encoded by AP1S2 in each patient, aiming to combine clinical data with gene regulation for analysis. The heatmap of the “IOD/Area” of each gene was plotted (**Figure 6B**). By implementing the median value of “IOD/Area,” 33 patients were divided into high and low groups, respectively. Consistently, Kaplan-Meier survival analysis indicated that GRM8 and AP1S2 expression was negatively correlated with prognosis (**Figure 6C**), which demonstrated that these two genes were specific indicators of poor diagnosis of Group 3 MB. However, with respect to MFAP2, the protein level was not significantly correlated with prognosis in our 33-patient series. Next, we collected detailed clinical data of the 33 patients in addition to postoperative survival time, such as sex, age, tumor cystic change, hydrocephalus, location of tumor body, tumor size, and Ki67 (%), to explore the deeper association of tumor phenotypes with the genetic hallmarks. However, most correlation coefficients were not statistically significant for GRM8, MFAP2, and AP1S2 (**Supplementary Tables 1–3**). Therefore, our study identified GRM8 and AP1S2 as two key regulators of Group 3 MB malignancy, which could serve as important biomarkers for Group 3 MB diagnosis and therapeutics.

## Discussion

Despite intensive conventional post-surgery treatments, half of the patients with Group 3 MB die from recurrent disease. *MYC* amplification and overexpression is known to play a vital role in maintaining the malignancy of Group 3 MB; however, an incomplete understanding of how MYC drives tumorigenesis in a subset of Group 3 tumor cells has hampered the development of novel therapeutic approaches for this lethal disease. The elucidation of novel critical factors regulating the malignant phenotype of MB cells is of great significance to increase our knowledge of this type of cancer, and subsequently bring more selective and efficient therapeutic options for patients with Group 3 MB.

Single-cell RNA sequencing allows us to understand how cellular heterogeneity contributes to the origination, progression, and invasion of Group 3 MB. MB single-cell RNA-seq datasets from the Gene Expression Database were analyzed in eight patients with Group 3 MB. Nine individual cell clusters were identified according to their distance distribution after dimensionality reduction through t-SNE. The genetic expression features of the cell clusters showed specific transcription preferences. Based on the cells’ pseudo-time, a trajectory analysis was performed showing the potent tumorigenesis characteristic of cluster 6 since cells congregated at the beginning of a differentiation tree. A differentiation trajectory might represent the degree of differentiation from a pluripotent cell to a terminal state (41). Therefore, cluster 6 is a potential tumorigenesis signature of Group 3 MB, and could be a foundation for further studies.

It has been shown that *MYC* amplifications are the most frequently observed driver events in Group 3 (42). In this study, PPI analysis showed that *MYC* is at the center of the network and crosstalks with critical downstream factors/targets pathways. Several immune system-related anti-tumor pathways such as antigen procession and presentation, and natural killer cell mediated cytotoxicity were specifically downregulated in cluster 6. Therefore, one potential therapeutic option for Group 3 would be to develop immunotherapy targeting the immune-related pathways and their correlated genes in cluster 6 cells, besides, to upregulate the immune response to tumor. This observation further clarifies the vital and specific role of cluster 6 cells in Group 3 MB.

Increasing evidence suggests that metabolic dysfunction is a key cause of cancer development, including MB. Interestingly, some amino acid synthesis pathways were enriched in cluster 6. Notably, previous studies have proven the important role of amino acids in cancer metabolism in both a tumorigenic and tumor-suppressive manner (43). These amino acid-related pathways specifically enriched in cluster 6 may be attributed to cancer formation and metastasis. Aminoacyl-tRNAs are substrates for translation, capable also of interacting with various proteins to regulate tumorigenesis (44). Consistently, proline and (or) arginine metabolism supports metastasis formation (40). In addition, cysteine is necessary to promote cancer cell proliferation and survival. The metabolic demands of a cell from the stresses associated with proliferation by oncogenic transformation must be met through extracellular sources of cysteine and *de novo* cysteine generation (45). Moreover, RNA polymerase (pol) III transcription contributes to the regulation of the cell’s biosynthetic capacity, and a direct link exists between cancer cell proliferation and deregulation of RNA pol III transcription (46). Further studies on the metabolism in Group 3 MB are warranted.

A pivotal role of cluster 6 has been identified and verified from several perspectives. Naturally, marker genes of this cell cluster are candidates for future therapeutic targets. From over thousands of genes, microarray data screening for survival and expression differences excluded most of them, only those prolonging or shortening survival while specifically expressed in MBs were selected. Therapy de-escalation of these genes requires prospective testing using clinical samples. Only GRM8, MFAP2, and AP1S2 were specifically upregulated in Group 3 MB; however, GRM8 and MFAP2 both interact with MYC as its downstream factors. The 33 cases of Group 3 MB provide us with valuable data showing that higher expression levels of GRM8 and AP1S2 are associated with poorer patient outcomes. All 33 patients pathologically diagnosed with Group 3 MB underwent craniotomy and achieved gross resection; adjuvant radiation therapy and chemotherapy were also performed. Thus, the correlation of the expression with survival is convincing to propose GRM8 and AP1S2 as novel hallmarks of Group 3 MB. Nevertheless, MFAP2 remains a potential tumor suppression feature based on previous results. However, the scale of the 33 clinical samples may be too small to uncover all associations.

GRM8 encodes MGLUR8, a G-protein coupled glutamate receptor reported to significantly influence the risk of diseases affecting the CNS including behavior, mental disorder, cognition as well as tumorigenesis of the CNS and other systems (28–30). Of note, GRM8 expression has been shown to characterize Group 4 MB (47) which overlaps in molecular features with Group 3 tumors. AP1S2 is a component of adaptor protein complex 1, and AP1S2 mutation could cause various brain diseases, including hydrocephalus and Dandy-Walker malformation, among others (31). To the best of our knowledge, this is the first study to reveal the functional significance of AP1S2 in Group 3 MB.

Clinical features such as cystic change, location, and tumor size have not been clearly related to molecular MB markers, or prognosis. Its large volume and cystic tumors can easily occlude the fourth ventricle causing hydrocephalus, while the location of the main tumor body is also important. Substantial Group 3 MBs originate and extend to the fourth ventricle, and obstructive hydrocephalus and cerebral fluid metastasis more often occur. In contrast, most SHH MBs are laterally located in the cerebral hemisphere; and therefore, hydrocephalus is not as common as Group 3 MB (48, 49). The Ki-67 index has been considered a valuable independent prognostic biomarker for adult MB (50), so we also included this index in the study. However, on comparing the above tumor features including age, sex, and hydrocephalus with the expression of GRM8 and AP1S2 within these 33 patients with Group 3 MB, no remarkable correlation between GRM8 and AP1S2 gene levels was found; therefore, a larger scale clinical sample is needed for further investigation.

## Conclusion

To summarize, using single-cell transcriptomics, this study identified GRM8 and AP1S2 as two novel hallmarks of Group 3 MB indicating poor prognosis, while simultaneously delineating a global picture of the molecular cascade of Group 3 MB downstream of the *MYC* oncogene. With the development of novel single-cell techniques combined with clinical cases, new insights into Group 3 MB molecular characteristics may promote clinical treatment and offer more selective and efficient therapeutic targets for this disease.

## Data Availability Statement

The datasets presented in this study can be found in online repositories. The names of the repository/repositories and accession number(s) can be found in the article/**Supplementary Material**.

## Ethics Statement

The studies involving human participants were reviewed and approved by Xiangya Hospital ethics committee. The patients/participants provided their written informed consent to participate in this study.

## Author Contributions

YP performed the experiments and interpreted the data. CQ wrote the manuscript. YuzL and YueL performed the experiments. WL and QL designed the experiments, interpreted the data, wrote the manuscript, and provided supervision. All authors contributed to the article and approved the submitted version.

## Funding

This work was supported by the National Natural Science Foundation of China (grant number 81802974).

## Conflict of Interest

The authors declare that the research was conducted in the absence of any commercial or financial relationships that could be construed as a potential conflict of interest.

## Supplementary Material

Supplementary Figure 1 CCND2, MPHOSPH6, TUBB4A, POU4F1, SLITRK1, KIF5C, and TSHZ1 mRNA levels of two normal brain tissues obtained from epilepsy surgery (Numbered 1 and 2), three Wingless/Sonic Hedgehog medulloblastoma (MB) (Numbered 3–5), and three Group 3 MB (Numbered 6–8). H-actin was used as endogenous reference gene. The expression level is presented with graphs.

## Abbreviations

MB, Medulloblastoma; WHO, World Health Organization; GRM8, Glutamate Receptor Metabotropic 8; MGLUR8, Metabotropic Glutamate Receptor 8; CNS, Central Nervous System; scRNA-seq, Single cell RNA sequencing; PDX, Patient-derived Xenograft; GSVA, Gene Set Variation Analysis; GO/KEGG, Gene ontology/Kyoto Encyclopedia of Genes and Genomes; PCR, Polymerase Chain Reaction; RT-Qpcr, Real Time Quantitative Polymerase Chain Reaction; IOD, Integrated option density; GSEA, Gene Set Enrichment Analysis; t-SNE, t-distributed Stochastic Neighbor Embedding.
