# Supplementary figures and images for: Novel Molecular Hallmarks of Group 3 Medulloblastoma by Single-Cell Transcriptomics
Source: Front Oncol. 2021 Mar 18;11:622430. doi: 10.3389/fonc.2021.622430 (PMC8013995; doi:10.3389/fonc.2021.622430)

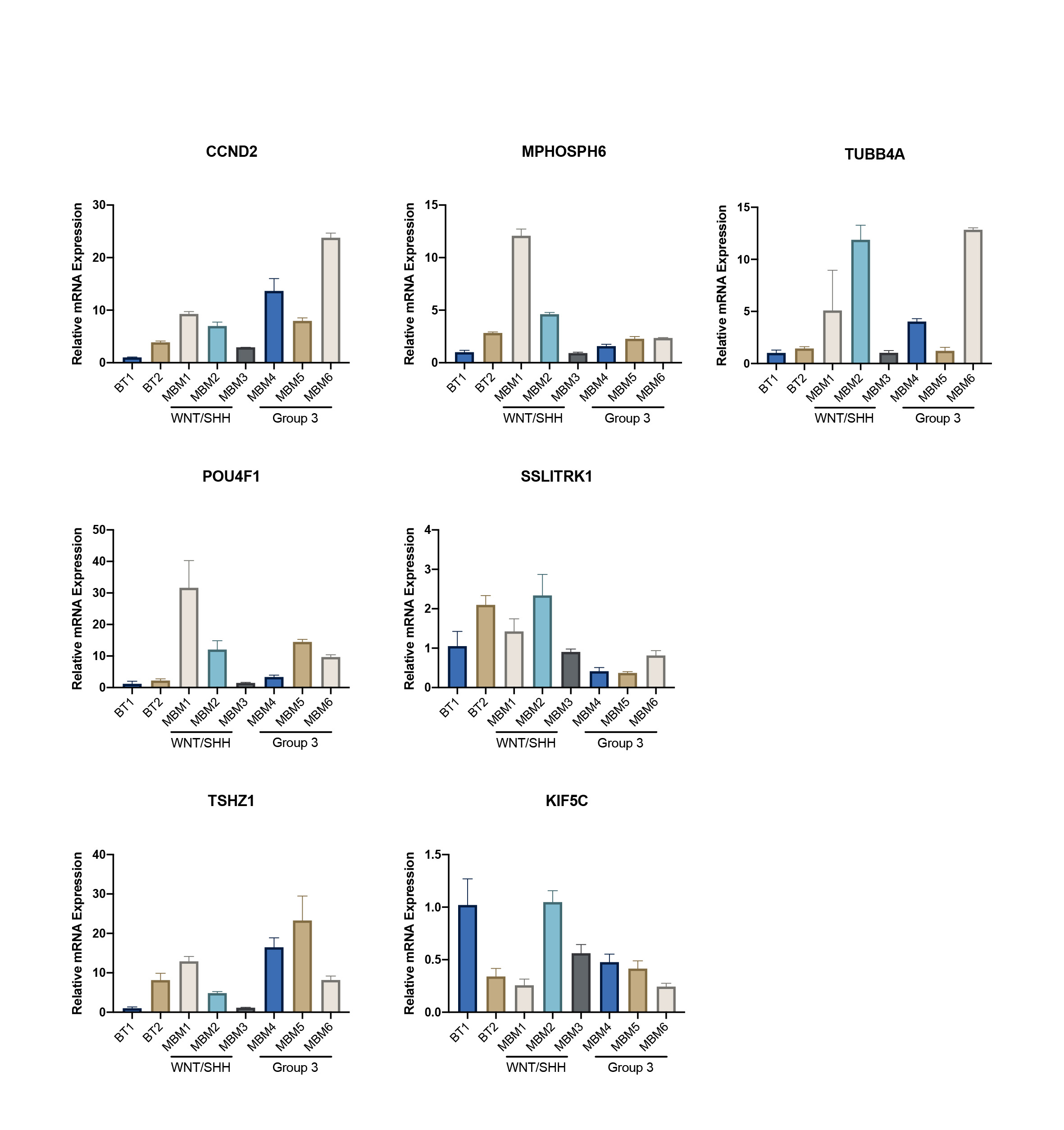

Supplement: Supplementary Figure 1 — CCND2, MPHOSPH6, TUBB4A, POU4F1, SLITRK1, KIF5C, and TSHZ1 mRNA levels of two normal brain tissues obtained from epilepsy surgery (Numbered 1 and 2), three Wingless/Sonic Hedgehog medulloblastoma (MB) (Numbered 3–5), and three Group 3 MB (Numbered 6–8). H-actin was used as endogenous reference gene. The expression level is presented with graphs. [file Image_1.jpeg]
