# Supplementary material for: Novel Molecular Hallmarks of Group 3 Medulloblastoma by Single-Cell Transcriptomics
Source: Front Oncol. 2021 Mar 18;11:622430. doi: 10.3389/fonc.2021.622430 (PMC8013995; doi:10.3389/fonc.2021.622430)
Supplement: Supplementary file 3 [file Table_2.docx]

**Table2.** Statistical analysis between clinical information and MFAP2 expression in MB patients

|  | **t-test**  （P-value） | **Univariable**  （P-value） | **Correlation coefficient** | |
| --- | --- | --- | --- | --- |
|  |  |  | （Spearman P-value） | （Pearson P-value） |
| **Gender**（Male/Female） | 0.192 | - |  |  |
| **Tumor cystic change**（Yes/No） | 0.043 | - |  |  |
| **Hydrocephalus**（Yes/No） | 0.330 | - |  |  |
| **Location** (Fourth Ventricle/Cerebellum/Both） |  | 0.501 |  |  |
| **Age** (years) |  |  | 0.102 | 0.945 |
| **Tumor size** (mm^3^) |  |  | 0.694 | 0.701 |
| **Ki67** (%) |  |  | 0.446 | 0.236 |
